# Lipid Nanocarriers for Hyperproliferative Skin Diseases

**DOI:** 10.3390/cancers13225619

**Published:** 2021-11-10

**Authors:** Eliana B. Souto, Ana L. R. de Souza, Fernanda K. dos Santos, Elena Sanchez-Lopez, Amanda Cano, Aleksandra Zielińska, Rafał Staszewski, Jacek Karczewski, Maria P. D. Gremião, Marlus Chorilli

**Affiliations:** 1 CEB—Centre of Biological Engineering, University of Minho, Campus de Gualtar, 4710-057 Braga, Portugal; 2 Faculty of Pharmaceutical Sciences, São Paulo State University (UNESP), Campus of Araraquara, Rodovia Araraquara Jaú, Araraquara 14800-903, SP, Brazil; alrsouza@ufg.br (A.L.R.d.S.); fernandakolenyak@yahoo.com.br (F.K.d.S.); palmira.gremiao@unesp.br (M.P.D.G.); marlus.chorilli@unesp.br (M.C.); 3 Department of Pharmacy, Pharmaceutical Technology and Physical Chemistry, Faculty of Pharmacy and Food Sciences, University of Barcelona, 08007 Barcelona, Spain; esanchezlopez@ub.edu (E.S.-L.); acanofernandez@ub.edu (A.C.); 4 Institute of Nanoscience and Nanotechnology (IN2UB), University of Barcelona, 08007 Barcelona, Spain; 5 Institute of Human Genetics, Polish Academy of Sciences, Strzeszyńska 32, 60-479 Poznań, Poland; aleksandra.zielinska@igcz.poznan.pl; 6 Department of Hypertension Angiology and Internal Medicine, Poznań University of Medical Sciences, 61-701 Poznań, Poland; rafal.staszewski@ump.edu.pl; 7 Department of Environmental Medicine, Poznań University of Medical Sciences, 61-701 Poznań, Poland; 8 Department of Gastroenterology, Dietetics and Internal Diseases, H. Swiecicki University Hospital, Poznań University of Medical Sciences, 60-355 Poznań, Poland

**Keywords:** lipid nanoparticles, liposomes, hyperproliferative skin diseases, antiproliferative drugs, skin cancer

## Abstract

**Simple Summary:**

Different drugs, including antiproliferative and corticosteroids in general, are recommended for the treatment of hyperproliferative skin diseases (HSD). The effectiveness of many of these drugs is limited due to their low solubility in water and low penetration in the skin. The loading of these drugs in lipid nanocarriers, such as lipid nanoparticles and liposomes, has been considered as a successful solution to improve the drug bioavailability through the skin, to control their release kinetics and thus reduce the risk of potential side effects. In this work, we discuss the use of lipid nanocarriers loading drugs against HSD.

**Abstract:**

Hyperproliferative skin diseases (HSD) are a group of diseases that include cancers, pre-cancerous lesions and diseases of unknown etiology that present different skin manifestations in terms of the degree and distribution of the injuries. Anti-proliferative agents used to treat these diseases are so diverse, including 5-aminolevulinic acid, 5-fluorouracil, imiquimod, methotrexate, paclitaxel, podophyllotoxin, realgar, and corticosteroids in general. These drugs usually have low aqueous solubility, which consequently decreases skin permeation. Thus, their incorporation in lipid nanocarriers has been proposed with the main objective to increase the effectiveness of topical treatment and reduce side effects. This manuscript aims to describe the advantages of using lipid nanoparticles and liposomes that can be used to load diversity of chemically different drugs for the treatment of HSD.

## 1. Introduction

Hyperproliferative skin diseases (HSD) do not include only the various types of cancers, as previously thought. They also relate to precancerous lesions and diseases of unknown etiology that can widely vary in the degree and distribution of the skin injuries, such as psoriasis, which can be caused by inflammatory responses [1,2]. Among the most commonly used drugs in the treatment of HSD, anti-proliferative agents, such as 5-aminolevulinic acid, 5-fluorouracil, paclitaxel, podophyllotoxin, methotrexate, imiquimod, and corticosteroids stand out [3].

**Figure 1:**
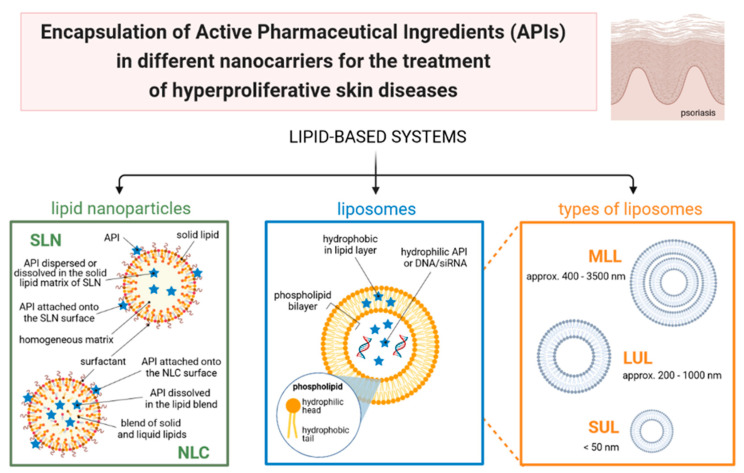
Schematic representation of the encapsulation of active pharmaceutical ingredients (APIs) in different types of lipid nanocarriers [own drawing].

The main limitations of these drugs are related to their physicochemical properties that hinder permeation through the skin [4,5,6]. The use of lipid nanocarriers (e.g., solid lipid nanoparticles (SLN), nanostructured lipid carriers (NLC), and liposomes), has been described as a successful approach to formulate these drugs (Figure 1) for the treatment of HSD to increase the efficacy of the topical treatment, in addition to reducing side effects [5,7].

The use of lipid carriers for the incorporation of drugs is not a recent approach. In 1965, Gregoriadis proposed for the first time the use of phospholipids in form of liposomes for drug delivery [8]. Amphiphilic molecules, as phospholipids, have a polar region (ionic) and an apolar region [9,10]. When present at a concentration higher than their Critical Micellar Concentration (CMC) and excess of water, can form different types of supramolecular aggregates, such as micelles, monolayers, multilayers and liposomes [9,11].

Liposomes are made up of one or more spherically arranged bilayers, separated by aqueous phases, encompassing an internal aqueous compartment [11,12]. These vesicles are organized in the presence of water, and, in part, the bilayer orientation is determined by the nature of the polar groups and carbon chains. Various amphiphilic compounds, containing two carbon chains, including the natural phospholipids and synthetic compounds [9,10], can be used as structural elements of liposomes while the production method can be fine-tuned to control the size and morphology of the vesicles [11].

Liposomes can be classified into three main groups, namely, multilamellar liposomes (MLL), small unilamellar liposomes (SUL), and large unilamellar liposomes (LUL) [12]. The MLL is formed by several concentric bilayers, interspersed with aqueous compartments. Their diameter is variable according to the number of lamellae, ranging approx. from 400 to 3500 nm. SUL, with a diameter <50 nm, consists of only a bilayer and a small watery compartment. LUL are also made up of only one bilayer, but with a large aqueous cavity. Their diameter ranges approx. from 200 to 1000 nm. Liposomes vary in size and homogeneity according to the production method and can be prepared by simply dispersing a film of amphiphilic molecules with mechanical agitation, sonication, reverse phase evaporation, extrusion, among others [13].

Most phospholipids do not form SUL spontaneously, requiring the supply of energy, for example, sonication [13]. During sonication, membrane fragments are formed, in which a hydrophobic part is exposed to the polar aqueous medium. When there is a transfer from an apolar medium to an aqueous medium, entropy is unfavorable. To overcome this, hydrophobic fragments join together. The unfavorable entropy of the interaction of the hydrophobic part of the fragments is equivalent to the unfavorable energy of the packaging. As a result, liposomes have a small radius of curvature surface. When energy is balanced, smaller liposomes are formed [14].

Liposomes are used as carriers for different molecules, offering a therapeutic improvement to loaded drugs, due to the high degree of biocompatibility and biphasic character [15]. The versatility of liposomes makes them suitable carriers for highly hydrophilic, highly lipophilic, and amphiphilic molecules.

The interaction of lipid nanocarriers with the skin is mainly governed by the nanocarriers’ structure which is defined by their lipid and surfactant composition. Potential alterations in the lipid domains of the stratum corneum were described for liposomes [16], whereas lipid nanocarriers with a solid matrix (e.g., solid lipid nanoparticles, nanostructured lipid carriers) are described to adhere onto the skin surface creating a lipid film that promotes drug permeation by increasing skin hydration [17]. The degree of drug permeation on the skin is, however, a matter of concern, whether a superficial (topical) or a deeper effect is expected without reaching systemic circulation (without transdermal delivery). The size and lipid composition are instrumental to control the extension of drug permeation in the skin. The presence of lipids able to disrupt the epidermis will promote a greater drug permeation [18]. 

Highly hydrophilic drugs or active pharmaceutical ingredients (APIs) are encapsulated in liposomes dissolved in the inner aqueous core. Lipophilic APIs will be encapsulated within the phospholipid bilayer, whereas amphiphilic APIs will be placed in between the aqueous core and lipid bilayers governed by their partition coefficient [15].

SLNs are considered promising carriers to modify the release profile of loaded APIs for the topical administration of drugs [19,20,21,22,23,24,25]. SLNs have a matrix composed of lipids that are solid at room and body temperature [17,26,27], being successfully used to improve the bioavailability of APIs by different administration routes, such as dermal, ocular, pulmonary, topical, among others [19,28,29]. SLNs have, however, some characteristics that limit their use, such as the low loading capacity due to the risk of expulsion of APIs from the matrix attributed to polymorphic changes of lipids during shelf-life, and also the high amount of water in the dispersions, which can vary from 70% to 90% [28,29,30].

To overcome the limitations of SLN, NLC has been developed [31,32]. The NLC matrix is composed of a mixture of a solid lipid and a liquid lipid that also melts above room and body temperatures thereby allowing to modify the release profile of loaded drugs [7,26,30,32]. The risk of polymorphic changes in the NLC matrix is reduced by the contribution of the liquid lipid, promoting a higher loading capacity when compared to SLN. Table 1 summarizes the main lipid nanocarriers that can be used in the treatment of HSD. Over the last few years, many studies have been published focusing on the topical application of lipid nanocarriers, highlighting their ability to load chemically different drugs to improve the bioavailability through the skin, by delivering them via controlled release kinetics, and showing no or low risk of systemic side effects or cytotoxicity [33,34]. Besides, lipid nanocarriers applied topically improve skin hydration attributed to their occlusive effects. Moisturized skin may also contribute to improved skin permeability of loaded drugs [35,36]. In this work, we discuss the background of HSD, such as basal cell and squamous cell carcinomas, skin melanoma and psoriasis, and how lipid nanocarriers can be an alternative over conventional treatments to improve drug bioavailability and reduce side effects. The selection of the lipid nanocarrier used in HSD is governed by the type of drug to be loaded (lipophilic versus hydrophilic), as these carriers are produced from biocompatible, biodegradable and biotolerable lipids that do not pose toxicological problems [33,34]. This aspect is instrumental for particles not only used in systemic treatments but also in topical treatments. While the purpose of using lipid nanocarriers to treat HSD is to increase the drug permeation/penetration into deeper skin layers, which is facilitated in diseased skin with compromised stratum corneum, for other topical applications (e.g., cosmetics) the drug has to remain onto healthy skin with minimal risk of permeating the skin.

## 2. Hyperproliferative Skin Diseases

### 2.1. Skin Cancer

Skin cancer is the general term used to refer malignant neoplasms which themselves refer to skin cells that have undergone a transformation and have multiplied in a disordered and abnormal way giving rise to a new tissue (neoplasia). Carcinoma refers to a cancer that begins in the epithelial tissue and can spread to other parts of the body. In the skin, one can describe the basal cell carcinoma, which is a slow-growing cancer that rarely spreads to other parts of the body. Squamous cell carcinoma also rarely spreads, but it occurs more frequently than basal cell carcinoma. These two types of carcinomas are sometimes called non-melanoma skin cancers. Another type of cancer that occurs on the skin is melanoma, which starts in the melanocytes, and all types of melanomas have the potential to cause metastasis if not previously treated [37].

The classification of a tumor as benign versus malignant is based on its capacity to originate metastases. Benign carcinoma is usually isolated, presenting as stable nodules, whereas the malignant lesion tends to invade other tissues, it grows rapidly and causes lesions with metastases. With the aging of the population, there is a simultaneous increase in actinic keratosis (pre-cancer lesions) and non-melanoma skin cancer, with solar radiation as the main cause. The cumulative effect of the sun can damage skin cells and lead to the development of skin cancer [37]. The oncogenic effects of solar radiation result from the action of electromagnetic waves on deoxyribonucleic acid (DNA). The formation of pyrimidine dimers in DNA by ultraviolet (UV) light, if not repaired, promotes errors in transcription that lead to the appearance of cancer.

UV radiation is primarily responsible for the development of skin cancer. Every time the skin is exposed to this radiation, either by direct sun exposure or through the use of artificial tanning techniques, lesions occur in the deeper layers of the skin that are not repaired. These lesions accumulate and, with systematic exposure to UV rays, they can end up producing malignant mutations, especially in people with fair skin. The keratinocytes of these patients are damaged by UV light, but the cells repair a large part of the DNA damage and the carcinoma only develops later. Thus, preventive measures of this disease from an early age, through the constant and rigorous use of photoprotection, is instrumental to reduce the risk of skin cancer [38].

In black people, much of this radiation is absorbed by the pigment melanin, not reaching the keratinocyte target; therefore, black skin does not normally develop basal cell and squamous cell carcinomas.

#### 2.1.1. Basal Cell Carcinoma

Basal cell carcinoma (basalioma or basal cell epithelioma) is the most common skin cancer, representing about 70% of all types. It originates in the cells of the basal layer of the epidermis and has a direct relationship with skin exposure to solar radiation, mainly to UVB radiation.

Basal cell carcinomas generally occur in individuals aged from 17 to 35 years [39]. They affect individuals with light skin, hair, and light eyes (Caucasians), mainly rural workers, and redheads and individuals with “freckles”. It usually appears after the 4th decade of life and is preferentially located in the areas of the body most exposed to the sun, such as face, neck, and back. Therefore, sun protection is the best way to prevent its appearance [38]. This tumor appears initially as reddish lesions on the skin that peel off and are slightly hardened. Its growth is very slow and gradual, not promoting metastases; however, they can turn into wounds that do not heal.

The vast majority of injuries appear on the face and in individuals over 40 years old. Its most typical presentation begins as a small, consistent lesion, pink or translucent in color and “pearly”, smooth and shiny, with thin blood vessels on the surface and grows progressively and slowly, giving rise to a nodule with subsequent central ulceration covered with a crust, which, when removed, leads to bleeding.

The treatment of basal cell carcinoma in the initial phase usually results in a 95% success rate of cure, being done through surgery (excision) or laser removal (cauterization). If it progresses without treatment, it can become very aggressive, invading the surrounding tissues and causing major defects and mutilations, especially in certain anatomical areas (nose, pinna, eyelids, etc.).

#### 2.1.2. Squamous Cell Carcinoma

Squamous cell carcinoma (squamous, squamous, or planocellular epithelioma) is a malignant tumor of the skin, characterized by variation in size and shape, hyper-colored and/or giant nuclei, loss of the nucleus-cytoplasm and nucleus-nucleolus relationship, frequently presenting mitoses. In the epithelium, the arrangement of cells is irregular and successive layers are not formed from the basal layer to the corneal layer; cornification is disordered and appears abruptly, off the surface and without normal progressive transformation.

Squamous cell carcinoma results from the atypical proliferation of spinous cells, of an invasive character, which can produce metastases. In addition, it usually appears after the age of 50. The characteristic lesions are nodules or plaques, reddish and thick, which bleed at the slightest trauma. The evolution of this carcinoma is fast, characterized by an increase in the size of these nodules, which may become verrucous or vegetating. Occasionally, they may develop lesions with high edges and a crusted surface on a high granular base.

This type of cancer can appear in areas of healthy skin or previously compromised by some other process, such as scars from old burns, chronic wounds, or injuries due to the skin exposure to solar radiation, such as actinic keratoses, which are caused by excessive accumulation of keratin in the epidermis [39]. Squamous cell carcinoma develops faster than basal cell carcinoma, reaching the skin and mucous membranes (oral mucosa and external genitalia) and can metastasize to other organs if not treated early.

The etiology of squamous cell carcinoma is multifactorial including, in addition to intense sun exposure, the use of immunodepressants (drugs used to prevent organ rejection after transplantation), exposure to industrial carcinogens (tar and oils), tobacco, among others [38]. The treatment of this carcinoma is usually surgical, through the total removal of the lesion (with control of the margins) and it must be carried out as early as possible to avoid the occurrence of metastases. Radiotherapy is also effective, especially when the location is in the lip.

#### 2.1.3. Melanoma

Melanoma consists of atypical melanocytes of the basal layer of the skin, with an invasive character that may result in metastasis to other regions of the body [40]. In addition, melanoma is most often located on the back, upper and lower limbs. It can appear spontaneously or can originate from a pre-existing signal [41].

Clinically, malignant skin melanoma is often asymptomatic, but the itching and bleeding wounds may represent the first manifestations of melanoma. Therefore, the most important clinical sign of a possible malignant degeneration is the color change in a pigmented lesion.

**Figure 2:**
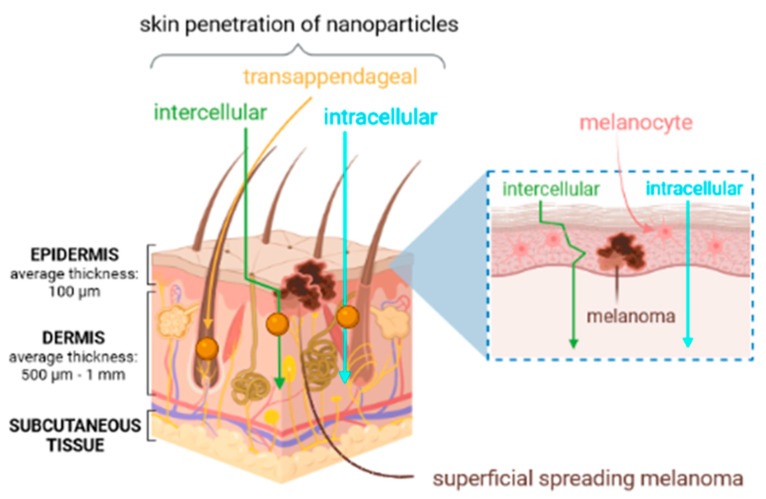
Different pathways of skin penetration of nanoparticles and superficial spreading melanoma (own drawing).

Many APIs have been proposed for the treatment of melanoma, but only a few show activity in more than 10% of patients [42]. The use of cytotoxic APIs remains the main form of chemotherapy for cancer. Cytotoxic APIs belong to a diverse class of compounds effective mainly because they are toxic to cells that are growing and dividing rapidly [43]. Figure 2 shows a schematic representation of superficial spreading melanoma and possible pathways of skin penetration of nanoparticles.

#### 2.1.4. Applications of Lipid Nanocarriers in Skin Cancer

Lipid nanocarriers have been proposed either for the protection of the skin from sun exposure (due to their inherent UV scattering effect) or for the delivery of chemotherapeutic drugs by topical administration [43,44].

5-fluorouracil (5-FLU), synthesized by Heidelberg et al. in 1957, is one of the oldest and most used chemotherapeutic drugs in the treatment of cancer [45]. It is an anti-metabolite of low molecular weight and easily soluble in water [43]. The effectiveness of 5-FLU was firstly described for the treatment of cancer whose lesions improved after systemic treatment with these APIs. Similar results were observed with its topical application in the treatment of actinic keratosis. Studies have shown that treatment with 5% 5-FLU improved lesions by 70% to 75% [45,46,47,48].

The photodynamic therapy (PDT) for the targeting of photosensitive drugs, such as 5-aminolevulinic acid (5-ALA), which is used to induce the production of protoporphyrin IX in cells [5,49,50], has also been exploited for the treatment of skin cancer. PDT is based on the use of a photochemical reaction between a light source of a specific wavelength and a photosensitizer in the presence of oxygen [51]. It is effective, safe, and has a long-term effect; however, because it has a hydrophilic precursor, it has a limited permeation through the skin.

PDT has been proposed in combination with lipid nanocarriers to overcome this limitation. Fang et al. described the degree of cutaneous penetration of 5-ALA in liposomes [52]. The authors observed that when encapsulated in liposomes, 5-ALA increased the production of protoporphyrin in the skin. Fadeel et al. proposed the use of polyethylene glycol-grafted (PEGylated) lipid nanocarriers loaded with curcumin to target skin cancer by photodynamic therapy [51]. Curcumin is an aromatic phytoextract obtained from the turmeric rhizome of *Curcuma longa*, with known anti-oxidant, anti-inflammatory and anti-cancer properties [53]. The poorly water-solubility of curcumin, however, limits its bioavailability in vivo. The loading of curcumin into lipid nanocarriers has been shown to improve the therapeutic effect of this polyphenol [54,55]. The human skin cancer cell line A431 was used to test the dark and photo-cytotoxicity of the curcumin-loaded PEGylated lipid nanocarriers, showing enhanced viability when cells were treated with the loaded curcumin when compared with the drug suspension [51]. By confocal laser microscopy, the in vivo studies in mice confirmed the increased permeation of curcumin, while histopathological analysis confirmed the presence of the drug in deeper layers of the skin after irradiation [51].

Palliyage et al. described the development of negatively charged solid lipid nanoparticles (SLN) for the dual loading of curcumin and resveratrol for the inhibition of proliferation of highly aggressive melanoma cells with high success [56]. Analysis by electrical cell-substrate impedance sensing suggested the potential blocking effect on cell migration of B16F10 melanoma cells by the dual therapy. A synergistic effect in inhibiting SK-MEL-28 melanoma cell proliferation was also described.

Temoporfin (mTHPC) is a potent second-generation synthetic photosensitizer with an effect on the photodynamic therapy of primary non-melanomatous tumors of the skin, head, and neck after intravenous administration topical formulations of mTHPC on the market is restricted [57]. Therefore, topical formulations containing mTHPC can be interesting in photodynamic therapy for both non-malignant diseases (psoriasis) or malignant skin diseases (basal cell carcinoma). However, mTHPC is a highly lipophilic drug with a molecular weight of 680 Da, which makes its skin permeation very low.

Dragicevic-Curic et al. described that deposition in the stratum corneum and accumulation in the deeper layers of the skin of mTHPC were greater for liposomes containing positively charged lipids. mTHPC was released into the stratum corneum and reached deeper skin layers in enough amount for topical PDT by administration of neutral or surface-loaded liposomes.

### 2.2. Psoriasis

Psoriasis is a chronic inflammatory disease of the skin and joints, autoimmune, with great polymorphism of clinical expression [58]. This disease is one of the most frequently described dermatoses in clinical practice, affecting men and women in the same way, characterized by cell renewal seven times faster than for normal skin, which is 28 days [7,59]. This disease can manifest itself in several different ways and presents itself in the form of minimal lesions to a more severe form, called erythrodermic psoriasis, in which the skin of the entire body may be compromised. However, the most frequent form of presentation is plaque psoriasis, characterized by the appearance of well-defined erythematous squamous skin lesions with chronic evolution. These scales are usually whitish and are most often located on the elbows, knees, scalp, and torso. Psoriasis lesions usually have no symptoms, but there may be mild itching at the site. When plaques regress, they usually leave a lighter area of skin in the affected area [60]. A metadata analysis published in 2019 reported the association between psoriasis and the risk of developing cancer [61].

Less common presentations are nail psoriasis, with lesions only on the nails, pustular psoriasis, with the formation of pustules mainly on the palms and soles of the feet, and psoriatic arthritis, which is characterized by joint inflammation that can even cause total destruction of the joint, usually affecting the fingers. The degrees of severity of psoriasis are divided into mild, moderate, and severe, one of the most serious being called inverse psoriasis, where the lesions are flat, inflamed, reaching large areas [62].

For some time, the question remained about the etiology of this pathology, whether it was just dermatological or immunological [63,64,65]. In this way, two possible theories have been proposed. The first assumes an unknown primary defect in keratinocytes that would produce cytokines if activated directly by physical or chemical trauma. These substances would activate T cells to release cytokines, with the consequent proliferation of lymphocytes and keratinocytes in the epidermis. The second theory deals with a mechanism secondary to the persistent activation of T lymphocytes by specific exogenous or endogenous factors (antigens, superantigens, or autoimmunity), leading to the proliferation and abnormal differentiation of keratinocytes [7,63].

The therapeutic strategy to be used in the treatment of psoriasis varies according to the conditions of each patient. The aim of the treatment is to decrease epidermal proliferation and the underlying inflammation, as there is no cure for this disease. Systemic treatment consists of using APIs such as cyclosporine, acitretin, and methotrexate, which can usually lead to hepatotoxicity and myelotoxicity. For topical therapy, the available treatment consists of the use of corticosteroids, calcipotriene, phototherapy with ultraviolet B radiation (UVB), among others [66,67].

The administration of drugs directly on the skin lesion makes it possible to minimize side effects on other organs and in the uninjured skin. In mild forms of psoriasis, topical therapy is usually enough to control the lesions. In moderate to severe forms, the association of local treatment with phototherapy and/or systemic therapy provides more comfort to the patient and accelerates skin recovery [7].

It is observed, therefore, that the APIs used in the treatment have serious side effects and research on new APIs for this purpose is limited. It is known that introducing a new active ingredient in the market, in addition to taking several years of research, involves very high costs. The alternative used to circumvent these high costs is to resort to the strategy of developing delivery systems for these active ingredients, which has allowed for an increase in their efficiency, the re-introduction of other active ingredients previously discarded for their undesirable properties, and the improvement of new substances before they are actually launched on the market or used in therapy [68].

Thus, in recent years, new drug delivery systems have been developed, such as lipid nanocarriers, with the objective not only of increasing the selectivity and effectiveness of active ingredients, but also allowing the reduction of its total necessary dose, minimizing toxic side effects, and allowing control of drug release [69,70].

#### Lipid Nanocarriers Applied in the Treatment of Psoriasis

Trotta et al. evaluated the permeation of methotrexate administered in phosphatidylcholine (PC) liposomes, hydrogenated phosphatidylcholine (HPC) and in deformable liposomes—PC or HPC liposomes containing a compound with emulsifying properties, dipotassium glycyrrhizinate—using dissected ears and hydrated pigs, maintaining the transepidermal hydration gradient [71]. The authors observed that the amount of methotrexate that permeated pig skin after a 24-h period was similar for the drug in aqueous solution or encapsulated in PC or HPC liposomes. However, when methotrexate was delivered in deformable liposomes, there was an increase in the amount of permeated drug. Drug penetration through the stratum corneum was facilitated when using deformable liposomes under the influence of transcutaneous hydration. The higher methotrexate permeation was attributed to the fusion of the deformable liposomes with the skin, facilitated by the increase in the fluidity of the phospholipid bilayers containing dipotassium glycyrrhizinate.

Another class of drugs used in the treatment of psoriasis is glucocorticoids [72]. Glucocorticoid receptors are located on viable keratinocytes and fibroblasts of the epidermis and dermis [73,74]. However, to reach these cells, drugs have to permeate the stratum corneum. The anti-proliferative and anthropogenic effects of steroids are due to the inhibition of IL-1α in fibroblasts; the anti-inflammatory effect results from the cytokine’s inhibition in keratinocytes [6,7,72].

Sivaramakrishnan et al. produced SLN by high-pressure homogenization, consisting of glyceryl behenate and poloxamer 188 (SLN A) or glyceryl and polysorbate 80 palmitoestearate (SLN B), for incorporation of 0.1% betamethasone valerate (BMV) [75]. The in vivo skin penetration studies of the different formulations (SLN A, SLN B, conventional cream containing 0.1% BMV, and nanoemulsion containing medium-chain triglyceride and poloxamer 188) were performed on the abdominal or breast skin of healthy humans. There was a fourfold increase in the penetration of BMV incorporated in SLN A compared to the control (cream with 0.1% BMV), which was not observed for BMV incorporated in SLN B and in nanoemulsions.

Zhang and Smith (2011) developed SLN containing BMV by the solvent injection method using monostearate or beeswax and lecithin as a surfactant as an oil phase [76]. In vitro skin permeation studies were carried out on Franz cells for the different formulations (lotions, suspensions, mono-stearic SLN, and beeswax SLN), in addition to assessing the distribution of corticosteroids and the formation of a reservoir in the skin. Lower BMV permeation flow [J = 0.1547 ± 0.009 µg/(cm^2^·h)] through the skin and greater APIs reservoir formation were observed for monostearate SLNs than for commercial lotions and APIs suspension, which can be advantageous for minimizing systemic absorption of APIs and, consequently, reducing the side effects that often occur when corticosteroids are absorbed into the bloodstream, in addition to increasing the amount of APIs at the site of action. In addition, the amount of BMV quantified in the epidermis and dermis was the highest when administered in form of monostearate SLN.

Fang et al. loaded 8-methoxypsoralen into Precirol and squalene composed nanostructured lipid carriers and demonstrated that drug entrapment minimized the permeation differentiation between normal and hyperproliferative skin compared to the free drug in nude mice [31].

Triamcinolone is an APIs widely used to relieve the inflammatory symptoms of corticosteroid-responsive dermatitis [77]. Yu and Liao (1996) developed ML and SUL containing triamcinolone and evaluated the effect of liposomal formulations on the skin permeation of these APIs [78]. The results were compared with the commercial triamcinolone ointment. The permeation study was performed on Franz cells using Wistar rat skin. The results obtained showed that the type of liposome (ML or SUL), its charges (positive, negative, or neutral), and the lipid composition influence the amount of APIs that permeates and is retained on the skin. Liposomes facilitated both the retention and cutaneous permeation of APIs. The permeability of triamcinolone was higher for liposomes than for conventional ointment. This in vitro study demonstrated that liposomes could increase the intradermal concentration of poorly water-soluble drugs.

Tretinoin is a metabolite of vitamin A that has also been widely used for the treatment of various inflammatory and proliferative skin diseases (e.g., psoriasis, acne, epidermotropic lymphomas originating from T-cells and epithelial cancer). Tretinoin plays a role in the regulation of epithelial cell growth and differentiation, sebum production and collagen synthesis [79]. However, because of its low water solubility, high instability in contact with air, light and heat, in addition to local skin irritation (risk of erythema, flaking and burning), the topical use of TRE is greatly limited [28].

To overcome these drawbacks, tretinoin has been formulated in delivery systems [80,81,82]. The loading of tretinoin in liposomal formulations reduced the undesirable effects of APIs on the skin’s surface, maximized its accumulation in the skin and prevented fast degradation. In vitro studies have reported an increase in the accumulation of tretinoin in the skin and in vivo studies have shown reduced irritation after liposomal treatment [83].

Acitretin, the second generation of aromatic retinoids, a metabolite of vitamin A, is another drug used in the treatment of psoriasis. This drug has the ability to establish a normal pattern of epithelial cell growth [84,85]. In a clinical study published by Agrawal et al. (2010), acitretin-loaded in NLC significantly reduced erythema compared to the conventional form [84]. The authors also noted a reduction in adverse effects, suggesting that treatment may be indicated primarily for patients who experience adverse reactions to topical psoriasis treatments.

Dithranol is highly effective for the treatment of psoriasis, but it is associated with irritation and necrotizing effects, burning and blemishes both on normal skin and on diseased skin [86]. Another substance reported in the literature is triptolide (diterpenoid triepoxide), a purified compound from traditional Chinese medicine extracted from a vine called *Tripterygium wilfondii Hook F*. Triptolide is quite efficient when absorbed into the bloodstream for the treatment of inflammatory and autoimmune diseases, and it presents immunosuppressive, antineoplastic and antifertility activities. Because of its low solubility in water and its adverse effects already reported in China, especially at the level of the gastrointestinal tract (e.g., nausea, vomiting, stomach pain, diarrhea, duodenal ulcer and hemorrhages), its clinical use is limited. Thus, the development of new triptolide delivery systems would lead to significant advantages in the clinical use of this drug [87,88].

Mei et al. (2005) developed SLN with triptolide and subsequent incorporation in Carbomer 940 hydrogels [88]. The in vitro skin permeation studies of triptolide from various dispersions of SLN and the hydrogel containing SLN were performed using the abdominal skin of rats with subcutaneous adipose tissue. Hydrogels with smaller particle sizes (tristearin SLN hydrogels) showed a greater amount of accumulated triptolide and prolonged release (during 24 h), in contrast to SLN dispersions, which released a greater amount of drug in the first 8 h. In addition, the percentage of transdermal absorption accumulated in 12 h was 45.3% for the conventional triptolide hydrogel, while for the SLN-triptolide hydrogel it was 73.5%, this percentage being determined by the diffusion of TP a from the solid lipid matrix to the hydrogel and, subsequently, to the skin. The formulation’s acute anti-inflammatory activity was evaluated in vivo in Wistar rats using carrageenan-induced paw edema as a parameter. The results obtained indicated that SLN-triptolide, hydrogels of SLN-triptolide and triptolide alone, showed acute anti-inflammatory activity, and the hydrogel of SLN-triptolide further reduced the paw edema induced by carrageenan when compared with SLN-triptolide and the control group. This anti-inflammatory effect was twice as high as the conventional triptolide hydrogel and especially better than that obtained for the control group, in which sodium diclofenac was used.

Tacrolimus (FK506) is an API with a well-tolerated immunosuppressive effect, extensively studied clinically for the treatment of chronic plaque psoriasis [89]. The most common adverse effects associated with its use include low and variable bioavailability, burning sensation, and itching at the application site, in addition to the potential to increase the risk of skin infections by altering the local immune response [90].

The in vitro permeation studies for tacrolimus were carried out with the use of the abdominal skin of hairless rats in Franz cells. They were performed by comparing the suspension of liposomes and liposomes incorporated in a Carbopol gel with the formulation of tacrolimus in propylene glycol, containing equivalent amounts of drugs [91]. With this study, it was found that there was a significant increase in the permeation of tacrolimus carried in the liposomes compared to the aqueous solution or Carbopol gel. The drugs permeated for 24 h were 65.71% and 61.45% from the liposomal suspension and liposome gel, respectively, while only 49.57% and 35.21% of the APIs permeated in the case of aqueous solution and Carbopol gel, respectively. Higher flow values obtained with the liposome suspension (2.623 ng/cm^2^/h) and liposome gel (1.740 ng/cm^2^/h) than those obtained with an aqueous solution (0.932 ng/cm^2^/h) and Carbopol gel (1237 ng/cm^2^/h) confirm the effect of APIs encapsulation in liposomes to improve its permeation. The phospholipid-rich domains of the vesicles may have helped to produce the deposition effect of APIs, reflecting a higher amount of drug retention in the skin layers in the case of liposomal formulations.

Photochemotherapy with psoralens is widely used in the treatment of psoriasis. The term PUVA, from the English “Psoralen UVA light therapy”, is used to define a group of therapeutic techniques that use psoralens and UV light. These are photosensitizing compounds derived from tricyclic furocoumarins, that occur naturally in some plants, or are produced by chemical synthesis. These compounds have a strong absorption band in the region of 200–350 nm. The main psoralens used in PUVA are 4,5′, 8-trimethylpsoralen (TMP), 8-methoxypsoralen (8-MOP) and 7-methylpyrido-(3,4-c)-psoralen (7-MOP), in combination with UVA radiation (PUVA therapy), which are used therapeutically in the treatment of proliferative skin disorders such as psoriasis and vitiligo.

Fang et al. developed SLN and NLC containing psoralens and examined the in vitro skin permeation of APIs, using hyperproliferative skin to mimic a clinical situation [52]. The permeation of 8-MOP followed zero-order kinetics for nanoparticulate systems and a biphasic release profile for the aqueous suspension. The flow of drugs through the rats’ skin was higher for NLC. The psoralens permeation increased in the following order: 8-MOP > 7-MOP > TMP.

Regardless of the type of HSD, the selection of the lipid nanocarrier is dependent on the type of drug (lipophilic versus hydrophilic), which will govern the selection of the lipid materials (fatty acids, mono-, di- or triglycerides, phospholipids, etc) in which the drug will show the highest solubility. The higher the solubility in the materials used in the production of the carrier, the higher the loading capacity and encapsulation efficiency. The increased encapsulation parameters will promote the capacity to reduce the dose required for a therapeutic effect and may thus reduce the risk of side effects.

Table 2 summarizes examples of APIs formulated into lipid nanocarriers for the treatment of hyperproliferative diseases.

## 3. Conclusions

The skin has numerous advantages both for topical and systemic administration of drugs. For the treatment of skin pathologies, such as hyperproliferative skin diseases, nanoparticles play a significant role in retaining the drug at the site of action and by modifying the release profile. The use of lipids resembling those existing in the skin structures in the production of lipid nanocarriers offers the additional advantage of being compatible and of creating a protective lipid film. The increased retention of antiproliferative drugs in the skin without inducing significant and irreversible changes in its barrier function opens the opportunity for developing new, more effective treatments for skin chemotherapy and psoriasis. Studies show that drugs used topically in HSD, when loaded into lipid nanoparticles, have a lower incidence of side effects and, particularly, demonstrate an increase in clinical efficacy. On the other hand, progress in particle engineering, in the design of new nanomaterials for the modified release of drugs, and a better understanding of how these particles interact with the (healthy versus damaged) skin will lead to significant advances in the treatment of hyperproliferative skin diseases.

## Figures and Tables

**Table 1:** Types of lipid nanocarriers proposed for the treatment of hyperproliferative skin diseases.

Systems	Description	Advantages	Limitations
Liposomes	Vesicles formed by lipid bilayers surrounding an aqueous core	Versatility to load lipophilic, hydrophilic and amphiphilic APIs	Low stability and lower loading capacity compared to SLN and NLC
SLN	Lipid nanoparticles composed of solid lipids with melting point above 40 °C	Possibility for scale-up production	Risk of API expulsion from the solid lipid matrix
NLC	Lipid nanoparticles composed of solid and liquid lipids with melting point above 40 °C	High loading capacity and encapsulation efficiency for lipophilic drugs; reduced risk of drug expulsion compared to SLN	Limited capacity to load hydrophilic APIs

**Table 2:** Examples of drugs loaded into lipid nanocarriers for the treatment of hyperproliferative diseases.

Drug	Type of Carrier	Mechanism of Action	Application	References
5-aminolevulinic acid	Oil-in-water emulsions	soybean oil o/w emulsions promoted mALA permeation to deeper skin layers	Treatment of subepidermal and subcutaneous lesions	[92]
	1,2-dipalmitoyl-sn-glycero-3-phosphocholine (DPPC) liposomes	Reduction of cell viability, mitochondria membrane potential, and enhancement of intracellular ROS accumulation	In melanoma xenograft models, 5-ALA/DPPC enhanced PpIX accumulation only in tumor tissue but not normal skin	[93]
	Liposomes based on mammalian stratum corneum lipids	Delivery of 5-ALA to viable epidermis and dermis	Photodynamic therapy of skin cancers	[94]
5-fluorouracil	Lecithin and poloxamer 188 based SLN	SLN-treated mice exhibited reduced inflammatory reactions, degree of keratosis, and symptoms of angiogenesis	skin carcinoma	[95]
	Tristearin, lecithin, polyvinyl alcohol and Tween 80-based SLN	Formulation development by quality by design		[96]
Guanosine -analogue phosphonate (OxBu)	SLN	Increased caspase-cleaved fragment of keratin-18, caspase-7 activation and reduced expression of matrix metallopeptidase-2 and Ki-67	Actinic keratosis, and cutaneous squamous cell carcinoma	[97]
Imiquimod	Vitamin D_3_-loaded monolithic lipid-polymer hybrid nanoparticles	In vivo efficacy assessment in imiquimod-induced psoriatic mouse model with enhanced anti-psoriatic activity	Imroved the Psoriasis Area and Severity Index (PASI)	[98]
	Chitosan Nanocapsules with Compritol 888 ATO	Imiquimod-loaded nanocapsules penetration into the stratum corneum and drug reached inner layers of the skin	Cell carcinoma, ac-tinic keratosis and genital and perianal warts	[99]
Methotrexate	Microemulsions in hydrogels	Location of drug at the desired domain of stratum corneum, epidermal and dermal layers of skin with reduction of systemic absorption	Successful treatment of imiquimod-induced psoriatic model, allergic contact dermatitis, rat tail model and safety	[100]
	Nanostructured lipid carrier	Reduction of PASI score with recovery of skin mice	Amelioration of symptoms of psoriasis imiquimod-induced psoriasis model	[101]
siRNA	Anti-STAT3 siRNA (siSTAT3) and anti-TNF-alpha siRNA (siTNF-alpha)-loaded in cationic amphiphilic lipid with oleyl chains- based nanoparticles	Efficient delivery of siSTAT3 and siTNF-alpha into the dermis	Combination of the two nucleic acids can synergistically treat psoriatic-like plaques	[102]
